# Biotechnologically Produced *Lavandula angustifolia* Mill. Extract Rich in Rosmarinic Acid Resolves Psoriasis-Related Inflammation Through Janus Kinase/Signal Transducer and Activator of Transcription Signaling

**DOI:** 10.3389/fphar.2021.680168

**Published:** 2021-04-27

**Authors:** Ivanka K. Koycheva, Liliya V. Vasileva, Kristiana M. Amirova, Andrey S. Marchev, Zhivka P. Balcheva-Sivenova, Milen I. Georgiev

**Affiliations:** ^1^ Laboratory of Metabolomics, Department of Biotechnology, The Stephan Angeloff Institute of Microbiology, Bulgarian Academy of Sciences, Plovdiv, Bulgaria; ^2^ Department Plant Cell Biotechnology, Center of Plant Systems Biology and Biotechnology, Plovdiv, Bulgaria

**Keywords:** psoriasis, keratinocytes, rosmarinic acid, inflammation, STAT1, *Lavandula angustifolia*

## Abstract

Psoriasis is a common skin pathology, characterized by dysregulation of epidermal keratinocyte function attended by persistent inflammation, suggesting that molecules with anti-inflammatory potential may be effective for its management. Rosmarinic acid (RA) is a natural bioactive molecule known to have an anti-inflammatory potential. Here we examined the effect of biotechnologically produced cell suspension extract of *Lavandula angustifolia* Mill (LV) high in RA content as treatment for psoriasis-associated inflammation in human keratinocytes. Regulatory genes from the nuclear factor kappa B (NF-κB) and Janus kinase/signal transducer and activator of transcription (JAK/STAT) signaling pathways were upregulated upon stimulation with a combination of interferon gamma (IFN-γ), interleukin (IL)-17A and IL-22. We also observed that both LV extract and RA could inhibit JAK2, leading to reduced STAT1 phosphorylation. Further, we demonstrated that LV extract inhibited phosphoinositide 3-kinases (PI3K) and protein kinase B (AKT), which could be implicated in reduced hyperproliferation in keratinocytes. Collectively, these findings indicate that the biotechnologically produced LV extract resolved psoriasis-like inflammation in human keratinocytes by interfering the JAK2/STAT1 signaling pathway and its effectiveness is due to its high content of RA (10%). Hence, both LV extract and pure RA possess the potential to be incorporated in formulations for topical application as therapeutic approach against psoriasis.

## Introduction

Psoriasis is a recurrent, chronic skin disease with complex immune-inflammatory ethology (Nestle et al., 2009; Sakurai et al., 2019). The worldwide prevalence of psoriasis is about 3% and the high societal burden of this condition is mainly due to the substantial impact on the patients’ perception of confidence and well-being (Lowes et al., 2007; Greb et al., 2016). Disrupted crosstalk between keratinocytes and immune cells is crucial determinant of chronic immune-mediated skin pathologies such as psoriasis (Federici et al., 2002; Nograles et al., 2008; Albanesi et al., 2018; Itoh et al., 2019). Cytokines and inflammatory factors such as interleukin (IL)-17A, IL-22, IL-23, and interferon gamma (IFN-γ), derived predominantly from dendritic cells and activated T cells, induce inflammatory response in keratinocytes (Wolk et al., 2006; Nograles et al., 2008; Albanesi et al., 2018; Liang et al., 2019). Consequently, activated keratinocytes respond with release of various cytokines (e.g., IL-1, IL-18, TNF-α), chemokines (CCL20), and antimicrobial peptides (S100A7, S100A8, etc.) that leads to additional recruitment of activated immune cells such as Th17 and contribute to the formation of a vicious cycle of psoriatic inflammation (Nograles et al., 2008; Shi et al., 2011; Lee et al., 2019). Therefore, modulating keratinocyte inflammation pathways can be targeted as an effective therapeutic approach in the management of psoriasis. Although the pathophysiology of psoriasis is complex and to a certain degree arguable, several important pathways are defined to play role in the progression of psoriatic inflammation, namely, activated nuclear factor kappa B (NF-κB), Janus kinase/signal transducer and activator of transcription (JAK/STAT), phosphoinositide 3-kinase/protein kinase B (PI3K/AKT) and mitogen activate protein kinase (MAPK) signaling (Engelman et al., 2006; Wolf et al., 2010; Andres et al., 2013; Schwartz et al., 2017; Li et al., 2019; Liang et al., 2019; Sakurai et al., 2019).

Currently available anti-psoriatic drugs include retinoids, corticosteroids and several immune-based drugs, which remain the main option for most patients with psoriasis (Lowes et al., 2007; Greb et al., 2016; Schwartz et al., 2017; Bigas et al., 2018). However, the adverse effects and possibility for development of drug-resistance are limiting the effectiveness of conventional anti-psoriatic therapy. Alternatively, natural products have substantial contribution in novel drug development owing to their biochemical diversity (Kim et al., 2014; An et al., 2018; Kim et al., 2018; Sun et al., 2019; Atanasov et al., 2021). Rosmarinic acid (RA) is a plant-derived secondary metabolite abundant in numerous plant species from the Lamiaceae and Boraginaceae families such as *Rosmarinus officinalis* L., *Salvia* spp., *Ocimum basilicum* L., *Perilla frutescens* L., *Origanum vulgare* L., *Melissa officinalis* L., and *Thymus vulgaris* L. (Petersen, 2013; Shen et al., 2017; Marchev et al., 2020). Significant pharmacological attention has been attracted toward RA as a result of its bioactive properties and, hence, therapeutic potential (Nunes et al., 2017; Luo et al., 2020). Among the wide range of its beneficial biological actions are antioxidant, anti-inflammatory (Chen et al., 2018; Joardar et al., 2019; Leu et al., 2019) anti-allergic (Liang et al., 2016), anti-fibrotic (Domitrovic et al., 2014; Joardar et al., 2019), neuroprotective (Shang et al., 2016) and anti-adipogenic (Vasileva et al., 2021). Apart from its potential medical applications, RA is also a preferred natural antioxidant and preservative in food and cosmetic industries (Kovatcheva-Apostolova et al., 2008; Shen et al., 2017; Chaiyana et al., 2019; Choi et al., 2019). Further, RA has been reported as an anti-ageing compound with applications in cosmetics (Shen et al., 2017; Chaiyana et al., 2019). More specifically, concerning RA effect on skin, several reports propose its anti-inflammatory and photo-protective properties (Sanchez-Campillo et al., 2009; Jang et al., 2011; Matwiejczuk et al., 2020). Data from our earlier studies pointed out RA as potent anti-inflammatory compound in IFN-γ-induced inflammatory skin condition (Georgiev et al., 2012). However, the underlying molecular mechanisms of RA in psoriasis-like inflammation in human keratinocytes are insufficiently explored.

Despite being a high-value molecule, its conventional extraction is economically impractical due to its low content, usually less than 1% of the dry weight (DW) of the source plant material (Petersen, 2013). Alternative approach for sustainable RA biosynthesis is the implementation of plant *in vitro* systems. Plant cell cultures give possibilities for the delivery of necessary amount of the targeted bioactive molecules, such as RA, regardless of the vegetative characteristics of the plant species or the limitation of the plant material and the climatic conditions (Goncalves et al., 2013; Marchev and Georgiev, 2020). We have previously reported the selection of high RA producing calli cell line PF of *Lavandula angustifolia* Mill (LV; Georgiev et al., 2006). Being a valuable aromatic plant *L. angustifolia* is predominantly used for distillation of essential oil. Therefore, the ethnopharmacological data regarding its use in psoriasis is limited to topical application of lavender oil. Human dermal fibroblast and endothelial cells exposed to *L. angustifolia* oil in the range of 0.016–2.00% (v/v) for 1 h responded with decreased cell viability in concentrations above 0.125% (Prashar et al., 2004). Another study evaluated the *L. angustifolia* oil in imiquimod-induced psoriasis-like model in mice. Topical application of 100 μL 10% lavender oil for four days resulted in anti-psoriatic activity comparable with that of 100 μg 1% mometasone cream used as positive control. In addition, the inhibition of inflammatory markers such as TNF-α, IL-1β, -17 and -22 (Rai et al., 2020). However, the potential application of the plant extract of *L. angustifolia* in skin inflammatory conditions such as psoriasis is insufficiently explored. The biotechnologically produced *L. angustifolia* calli culture (cell line PF) was selected for its high biosynthetic capacity that was reported to be stable upon 5 to 10 cultivations, producing RA 1.7-folds more that in the control culture (Georgiev et al., 2006). The capacity of the LV cell line for RA biosynthesis, as well as quantification of the RA yield with contemporary phytochemical analytical techniques, has not been evaluated to date. Confirmation of the high productivity in LV cell line reported 15 years ago would provide significant evidence for the stability of this plant *in vitro* culture as biotechnological matrix for RA large-scale utilization.

In the present study, we examined the content of RA in biotechnologically produced LV extract in order to evaluate whether this *in vitro* culture have preserved its high production capacity after 15 years of maintenance. Additionally, we have investigated the anti-inflammatory potential of LV extract and corresponding concentrations of RA on *in vitro* psoriatic-like model in human keratinocytes, HaCaT cell line. Further, the underlying signaling pathways involved have been investigated.

## Materials and Methods

### Materials

Deuterated methanol (purity 99.8%) and deuterium oxide (99.9%) were supplied from Deutero GmbH (Kastellaun, Germany). Dulbecco’s Modified Eagle Medium (DMEM) with high glucose 4.5 g/L (#D5796), fetal bovine serum (FBS; #F7524), 3-(4,5-dimethyl-2-thiazolyl)-2,5-diphenyl-2H-tetrazolium bromide (MTT; #M21281), penicillin/streptomycin/amphotericin B (10,000 IU/10 mg/25 μg; #A5955), trypsin-EDTA (#59418C), dexamethasone (DEXA; purity ≥98%; #D1756), rosmarinic acid (purity ≥98%, #R4033), trimethyl silylpropionic acid sodium salt-d4 (TSPA-d4; #11202), methanol, acetic acid of HPLC grade, Bradford reagent (#B6916), RIPA lysis buffer (#R0278), protease and phosphatase inhibitor cocktail (#PPC1010) and RNAzol RT reagent (#R4533) were from Merck KGaA (Darmstadt, Germany). The recombinant human IL-17A (#ENZ-PRT188) and IL-22 (#ENZ-PRT250) were purchased from Enzo Life Sciences AG (Lausen, Switzerland) and IFN-γ (#10067-IF) was obtained from R&D Systems (Minneapolis, MN, United States).

Buffers and chemicals used to perform electrophoresis, immunoblotting and real-time quantitative reverse transcription-polymerase chain reaction (RT-qPCR) were obtained from Bio-Rad Laboratories, Inc (Hercules, CA, United States). For the Western blotting analysis primary antibodies against the following proteins were used: AKT (#9272), JAK2 (#3230S), PI3K (#4257), STAT1 (#14994) and phospho-STAT1 (#7649) from Cell Signaling Technology (Leiden, Netherlands); tubulin (#12004166) from Bio-Rad. Secondary anti-rabbit IgG Star-Bright Blue 700 (#12004162) antibody from Bio-Rad was used for fluorescent detection. All other materials and substances were of analytical grade and were purchased from Merck KGaA (Darmstadt, Germany) unless otherwise specified.

### Cultivation of *Lavandula angustifolia* Cell Suspension and Extraction Procedure

The LV suspension culture was cultivated as previously reported on liquid Linsmayer and Skoog (LS) nutrient medium supplemented with 0.2 mg/L 2,4-dichlorophenoxyacetic acid and 30 g/L sucrose in darkness at 26°C on a rotary shaker at 100 rpm (Georgiev et al., 2006). Following cultivation, the cell suspension was freeze dried and extracted with 50% aqueous methanol under sonication at room temperature (RT) for 20 min. Further, the extract was filtrated, vacuum concentrated at 40°C, lyophilized and stored at −20°C until use.

### Phytochemical Analysis

Nuclear magnetic resonance (NMR) analysis was performed according to the protocol described by (Georgiev et al., 2015). Sample of 10 mg of the obtained LV extract was dissolved in equal amounts of 0.4 ml CD_3_OD and D_2_O, containing KH_2_PO_4_ buffer with pH 6.0 and TSPA-d4 as internal standard at final concentration of 0.005% (w/v). ^1^H NMR and 2D: J-resolved, homonuclear correlation spectroscopy (COSY) and heteronuclear single quantum coherence spectroscopy (HSQC) spectra were obtained on a Bruker AVII+ 600 spectrometer (Bruker, Karlsruhe, Germany), operating at 25°C, 600.13 MHz proton frequency, with 4.07 s relaxation time and CD_3_OD as an internal lock.

Quantification of the RA content in the extract was executed with high-performance liquid chromatography (HPLC). Reference RA standard was dissolved in methanol and standard solutions from 10–200 μg/ml were prepared and filtrated through 0.45 µm syringe filters for standard curve preparation. The suspension extract was prepared in 5 mg/ml solutions in 50% aqueous methanol. The analyses were performed on Waters HPLC system (Milford, MA, United States), consisting of binary pump and dual wavelength (λ) absorbance detector. A reverse-phase Kinetex^®^ C18, 100 Å (150 × 4.6 mm, 5 μm) core-shell column (Phenomenex, Torrance, CA, United States), operating at 26°C was used. The RA determination was based on an HPLC protocol previously used by Choi et al. (2019) with certain modifications. The mobile phases used were 0.02% aqueous acetic acid (phase A) and methanol (phase B) at flow rate of 0.8 ml/min with the following gradient: change of phase A from 88 to 85 (0–5 min), from 85 to 82 (5–6 min), from 82 to 75 (6–9.5 min), from 75 to 74 (9.5–10.5 min), from 74 to 73 (10.5–12 min), from 73 to 70 (12–20 min), from 70 to 50 (20–25 min), from 50 to 30 (25–30 min) and from 30 to 88 (30–35 min) at λ of 320 nm.

The HPLC method was validated for linearity and sensitivity, including limit of detection (LOD) and limit of quantification (LOQ). An external standard calibration methodology was applied. Seven solutions with different concentrations ranging from 10 to 200 μg/ml were prepared. The analyses were performed in triplicates (for each concentration) and the calibration curve was constructed as a relationship between the peak areas and the used concentrations of the standard. The linearity of the curve was assessed according to the obtained regression equations y = ax + b, where y and x refer to peak area and the concentration of the compound (in μg/ml) and its relevant coefficient of determinations (R^2^). The sensitivity was determined by calculating the LOD = 3.3σ/S and the LOQ = 10σ/S, where σ is standard deviation of the response and S is the slope of the calibration curve.

### Cell Culture and Treatment

Human epidermal keratinocytes (HaCaT) cell line was purchased from Cell Line Service GmbH (Eppelheim, Germany). The HaCaT cells were cultured in DMEM with 4.5 g/L glucose supplemented with 10% FBS and 1% antibiotic and anti-mycotic solution and maintained at 37°C with 5% CO_2_. At 80% confluence HaCaT cells were stimulated with combination of IFN-γ/IL-17A/IL-22 (1 ng/ml each) to model psoriasis-like inflammatory milieu 1 h prior treatment with either LV (20, 40, 100 μg/ml), RA (5, 10, 25 µM), vehicle alone (0.02% DMSO) or DEXA 5 µM (as a positive control). Total RNA samples were obtained 6 h after treatment and whole cell protein lysates on the 24th hour following treatment.

To determine the effect of LV and RA at different concentrations on cell viability in HaCaT, MTT assay was conducted. Exposure to LV and RA in HaCaT cells for 24 h did not affect significantly the cell viability up to 100 μg/ml and 100 μM, respectively (Supplementary Figure S1).

### Quantitative Real-Time Polymerase Chain Reaction

Total RNA was isolated using RNAzol RT reagent and reverse transcribed using FirstStrand cDNA kit (#PR008) from Canvax (Cordoba, Spain) according to the manufacturer’s instructions. The relative expression of target genes was detected by RT-qPCR using Sso EvaGreen SuperMix (#1725204; Bio-Rad) on CFX96 detection sys-tem (Bio-Rad) and was quantified by the comparative threshold cycle (_ΔΔ_CT) method on the CFX Maestro software (Bio-Rad). The primer sequences are listed in Supplementary Table S1. Both *GAPDH* and *TUBB* were used as reference genes and served for normalization of all the other gene expression.

### Western Blotting

Total protein lysates were prepared using RIPA buffer freshly supplemented with 1% protease and phosphatase inhibitor cocktail for 15 min on ice, followed by centrifugation (15,000 rpm, 15 min, and 4°C) and collection of the clear supernatant. The total protein concentration was determined using Bradford assay.

Equal amounts of total protein of 50 μg per lane were separated with vertical electrophoresis on 10% TGX Stain-Free SDS-PAGE gels and transferred to nitrocellulose membranes *via* semi-dry transfer with *Trans*-Blot Turbo transfer system (Bio-Rad). Following blocking for 1 h at RT in 5% (w/v) skimmed milk in Tris buffered saline, the membranes were incubated with the primary rabbit antibodies against AKT, JAK2, PI3K, STAT1 and p-STAT1 overnight at 4°C. For fluorescent detection of the target proteins Star-Bright Blue 700 goat anti-rabbit IgG secondary antibody was used. Tubulin was detected with direct primary antibody as a housekeeping protein used for normalization. The immunodetection was visualized with ChemiDoc MP imaging system (Bio-Rad) and the acquired images were analyzed with Image Lab software 6.0.1 (Bio-Rad).

### Statistical Evaluation

The obtained spectra were automatically reduced to ASCII files using AMIX soft-ware (version 3.7, Bruker), phased, base line corrected and referenced at 0.0 ppm to the internal standard TSPA-d4 using MestReNova software (version 12.0.0, Mestrelab Research, Santiago de Compostela, Spain). All signals were normalized in relation to the peak of TSPA-d4 and scaled to 1.0.

Data evaluation was performed with SigmaPlot software v11.0 (Systat Software GmbH, Erkrath, Germany) and the results are presented as mean ± standard error of the mean (SEM). Differences among groups was determined with unpaired Student’s *t*-test as appropriate or one-way analysis of variances (ANOVA) with Bonferroni’s post hoc test when more than two groups were compared. The significance level was defined as + *p* < 0.05, model group compared to non-treated control cells, *p < 0.05 and **p < 0.01, compared to IFN-γ/IL-17A/IL-22-stimulated model group.

## Results

### Phytochemical Analysis of *Lavandula angustifolia* Cell Suspension Extract

Nuclear magnetic resonance (NMR) phytochemical profiling has a great potential for studying living organisms, owing to its non-destructive nature, *i.e.* it can be used for *in vivo* and *in vitro* measurements of biological processes, with no quenching of the metabolism required. The investigated extract in our study was obtained from high RA producing *L. angustifolia* cell suspension induced and maintained within our lab (Georgiev et al., 2006). The phytochemical characterization has been performed by 1D- and 2D-NMR metabolite profiling. According to the ^1^H NMR spectral data the most abundant signals observed in the aromatic region corresponded to RA as a major secondary metabolite. Further, metabolites typical for the primary cell metabolism were identified as well. In the aliphatic region (*δ* 0.5–3.0 ppm) the signals of several organic acids (acetic, formic, fumaric and pyruvic acids) were detected. The identified metabolites are important intermediates from the tricarboxylic acid cycle (TCA), which is a main source of energy for cells and important part of erobic respiration. The TCA is a part of a larger carbohydrate metabolism in which the metabolites identified in the carbohydrate region (*δ* 3.0–5.5), such as α-, β-glucose and sucrose are oxidized to form pyruvate. The latter is an entry metabolite of the TCA cycle under the form of acetyl-CoA. The TCA cycle is also the origin of pathways leading to important structural metabolites, including the amino acids alanine, glutamine, glutamate, threonine and valine, identified in the extract (Table 1).

**TABLE 1:** Chemical shifts (*δ*) and coupling constants (*J*) of the metabolites, identified in *Lavandula angustifolia* extract by their relevant ^1^H NMR spectra (Pereira et al., 2013; Georgiev et al., 2015; Marchev et al., 2017).

Metabolite	Chemical shift (ppm)	Coupling constant (Hz)
**Amino acids**		
Alanine	1.49	(d, *J* = 7.3)
Glutamine	2.14/2.37	(m)/(m)
Glutamate	2.37/2.06	(m)/(m)
Threonine	1.33	(d, *J* = 6.8)
Valine	1.01/1.06	(d, *J* = 7.1)/(d, *J* = 7.1)
**Carbohydrates**		
α-Glucose	5.19	(d, *J* = 3.7)
β-Glucose	4.59	(d, *J* = 7.9)
Sucrose	5.41/4.18	(d, *J* = 3.9)/(d, *J* = 8.9)
**Organic acids**		
formic acid	8.48	(s)
acetic acid	1.91	(s)
Fumaric acid	6.53	(s)
Pyruvic acid	2.43	(s)
**Phenolic acids**		
Rosmarinic acid	7.12/6.88/7.04/7.52/6.32/6.84/6.78/6.72/2.94/3.11/5.03	(d, *J* = 2.1)/(d, *J* = 8.2)/(dd, *J* = 8.4, 2.1)/(d, *J* = 15.9)/(d, *J* = 15.9)/(d, *J* = 2.0)/(d, *J* = 8.1)/(dd, *J* = 8.1, 2.0)/(dd, *J* = 14.4, 10.0)/(dd, *J* = 14.5, 3.5)/(dd, *J* = 10.0, 3.4)
**Others**		
Choline	3.22	(s)
Pyruvic acid	2.43	s
γ-Amino-butyrate (GABA)	1.92/2.30/3.01	(m)/(t, *J* = 7.2)/(t, *J* = 7.3)
Inosine	8.24/8.35	(s)/(s)
Inositol	3.95/3.52/3.21	(m)/(m)/(m)

Rosmarinic acid [(R)-a-[[3-(3,4-dihydroxyphenyl)-1-oxo-2E-propenyl]oxy]-3,4-dihydroxy-benzenepropanoic acid] is a polyphenolic compound, an ester of caffeic acid and 3-(3,4-dihydroxyphenyl)-lactic acid. Its structure (C_18_H_16_O_8_) comprises of two unsaturated six-membered rings (C1-C6) and (C1′-C6′), a double bond (C7′-C8′), an ester group (C9′), a carboxyl group (C9) and four hydroxyl groups (C3, C4, C3′ and C4’; Xiao et al., 2008). In the current investigation, the RA was structurally identified by its 1D and 2D NMR spectra and quantified using high performance liquid chromatography (HPLC). The signals from the proton spectrum (Table 1) of RA identified in the extract were in accordance with the signals previously reported (Pereira et al., 2013; Zhao et al., 2021).

**FIGURE 1:**
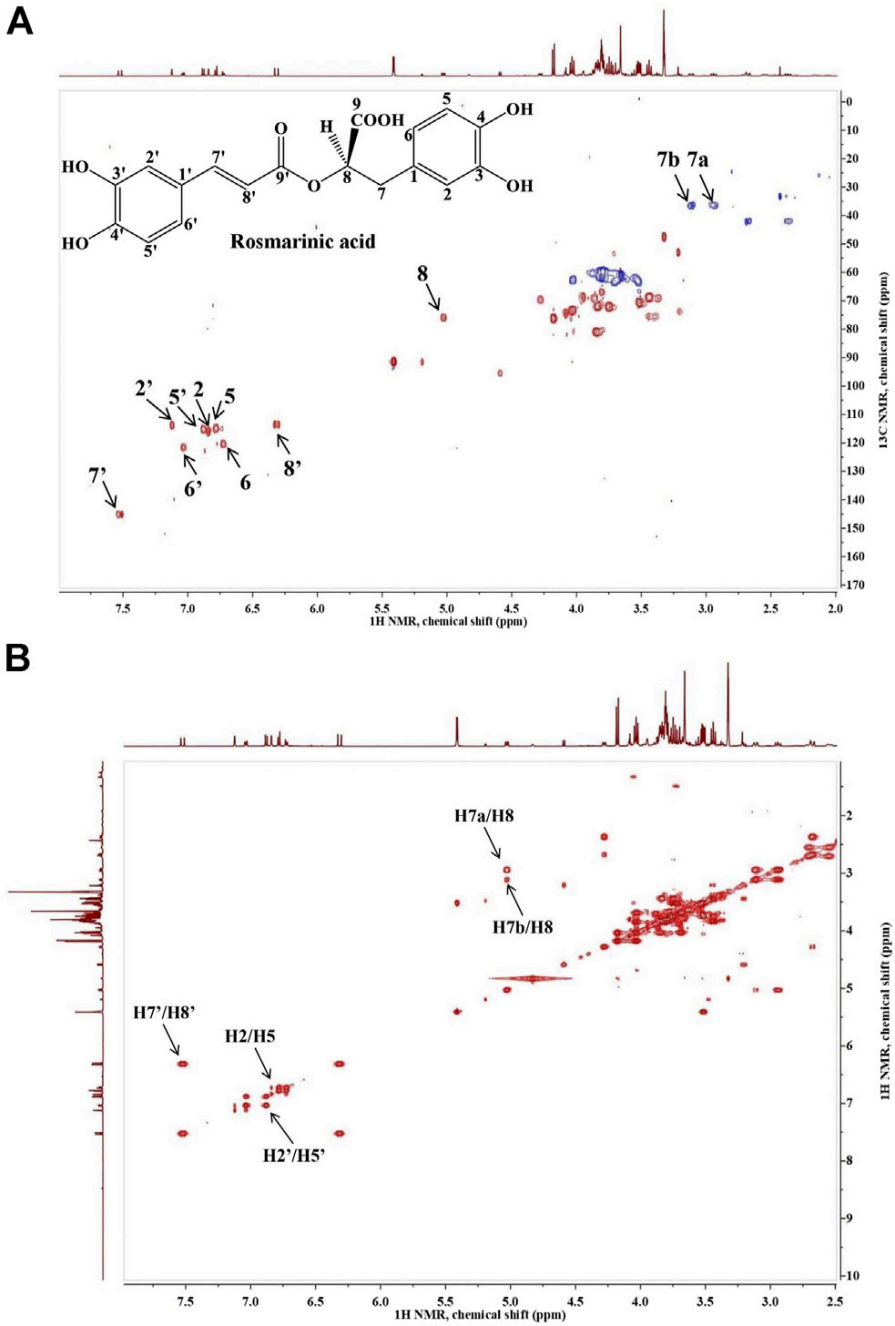
Heteronuclear single quantum coherence spectroscopy (^1^H-^13^C HSQC **(A)**) and homonuclear correlation spectroscopy (^1^H–^1^H COSY **(B)**) spectra of *Lavandula angustifolia* extract and the characteristic signals of rosmarinic acid.

The presence of RA in the analyzed extract was confirmed by the proton-carbon single bond correlations observed in the HSQC spectrum, shown on Figure 1A. The spectrum of the extract revealed the presence of two doublets *δ*_*H*_ 7.52/*δ*_*C*_ 145.14 and *δ*_*H*_ 6.32/*δ*_*C*_ 113.36, which on basis of the large proton-proton coupling were assigned to a pair of *trans*-olefinic protons (H-7′ and H-8′), indicating the presence of E-caffeic acid moiety. Further, the ABX-system of caffeic acid was confirmed by the three protons H-5′, H-6′ and H-2′, which referred at *δ*_*H*_ 6.88/*δ*_*C*_ 115.21, *δ*_*H*_ 7.04/*δ*_*C*_ 121.59 and *δ*_*H*_ 7.12/*δ*_*C*_ 113.84, respectively. The other ABX-system in the aromatic region was assigned to the 3,4-dihydroxypehenyl unit according to the three protons H-5, H-6 and H-2, which appeared at *δ*_*H*_ 6.78/*δ*_*C*_ 114.87, *δ*_*H*_ 6.72/*δ*_*C*_ 120.61 and *δ*_*H*_ 6.84/*δ*_*C*_ 116.02. The doublet in the low field region at *δ*_*H*_ 5.03/*δ*_*C*_ 75.84 suggested a proton of a CH group attached to an oxygen-bearing carbon was assigned to H-8. Two doublets at *δ*_*H*_ 2.94/*δ*_*C*_ 36.36 and *δ*_*H*_ 3.11/*δ*_*C*_ 36.36 were assigned to the two protons of 7-methine group. In addition, on Figure 1B is shown the long-rage ^1^H–^1^H COSY interactions. Strong cross-peaks were found between H-7’/H-8’ (*δ*_*H*_ 7.52/*δ*_*C*_ 6.32), H2’/H-5’ (*δ*_*H*_ 7.12/*δ*_*C*_ 6.88) and H2-H-5 (*δ*_*H*_ 6.84/*δ*_*C*_ 6.78; Xiao et al., 2008; Pereira et al., 2013; Zhao et al., 2021).

The HPLC method used to detect and quantify RA was validated for linearity and sensitivity. The obtained calibration curve (y = 7.96 × 10^4^x-3.31 × 10^5^) had a good linear relationship in the concentration range between 10 and 200 μg/ml, characterized with R^2^ of 0.9918. The LOD and LOQ values, 6.9 and 14.59 μg/ml, respectively also indicated a satisfactory sensitivity.

According to the HPLC quantification, the content of RA in the cell suspension resulted to 100.16 ± 7.01 mg/g extract.

### *Lavandula angustifolia* Extract and Pure Rosmarinic Acid Diminished Psoriasis-Associated Inflammatory Changes in Gene Expression Profile in Keratinocytes

The psoriatic condition is characterized by activation of inflammatory signaling pathways. The NF-κB pathway is a key regulatory pathway during inflammation, and is considered to be a crucial mediator in the pathogenesis of psoriasis (Andres et al., 2013; Kim et al., 2014; Zhang et al., 2018). Additionally, JAK/STAT pathway is a classical signal transduction pathway in psoriasis and is triggered upon cytokine stimulation (Shi et al., 2011; Schwartz et al., 2017; Sun et al., 2019). Activation of the p38/JNK/ERK MAPKs (Shi et al., 2018; Liang et al., 2019; Sakurai et al., 2019) and PI3K/AKT (Engelman et al., 2006; Li et al., 2019) signaling pathways contributes to psoriasis exacerbation and disturbed regulation of proliferation and apoptosis in activated keratinocytes.

**FIGURE 2:**
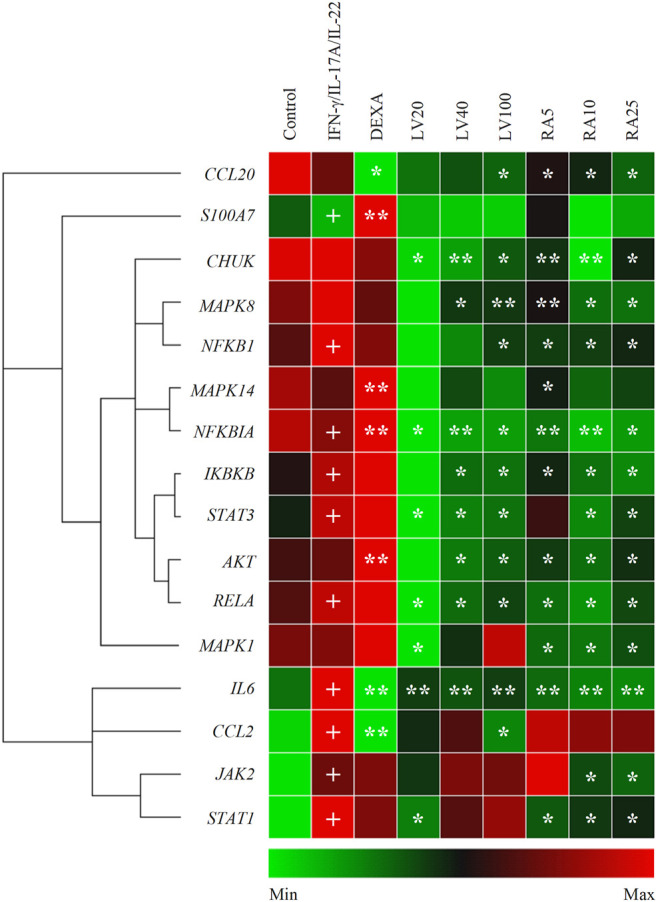
*Lavandula angustifolia* extract (LV) and pure rosmarinic acid (RA) downregulate inflammatory gene expression in psoriasis-like model in human keratinocytes. Clustergram and heatmap of the relative gene expression analysis from the RT-qPCR. The results are expressed as mean ± SEM from three independent experiments. +*p* < 0.05 compared to control cells; *p < 0.05 and **p < 0.01 com-pared to psoriasis-like model group.

Stimulation with IFN-γ/IL-17A/IL-22 cytokine combination induced changes in the mRNA expression profile in human keratinocytes that mimic psoriasis-like transcriptional signature (Figure 2). Data from the hierarchical cluster analysis of the relative gene expression obtained with RT-qPCR indicated significant upregulation of genes related to NF-κB, JAK/STAT, p38/MAPK/AKT signaling pathways, as well as pro-inflammatory cytokines. The inflammatory factors *IL6* and *CCL2* (encoding MCP1 protein) were highly overexpressed as a result of the combined psoriasis-induction stimuli that reflects acute inflammation changes. In contrast, the anti-bacterial peptide *S100A7* and the chemokine *CCL20* that are frequently used as markers of psoriasis were moderately changed in the model group. The mRNA expression of *NFKB1*, *NFKBIA*, *IKBKB* and *RELA* was elevated in the model group, hence, suggesting activation of the NF-κB signaling pathway. Furthermore, overexpression was detected for the *JAK2*, *STAT1* and *STAT3* genes, which is in agreement with previous reports utilizing similar combinatorial stimulation used for modeling psoriasis in keratinocytes (Kim et al., 2014; An et al., 2018; Zhang et al., 2018; Li et al., 2020). The increase in *STAT1* was about 10 folds higher that the overexpression of *STAT3* upon stimulation with IFN-γ/IL-17A/IL-22, hence, pointing out *STAT1* as the more influenced in our model.

Treatment with LV extract dose-dependently downregulated *IL6*, *CCL2* and *CCL20*. Further, the inhibition of mRNA expression of genes from the NF-κB signaling (*CHUK*, *NFKB1*, *NFKBIA*, *IKBKB*, *RELA*) at the highest treatment concentration of LV (100 μg/ml) exceeded that achieved with the positive control DEXA 5 μM. The profound influence of the LV extract on the NF-κB-related genes suggest its potent anti-inflammatory action in psoriatic keratinocytes. Additionally, the key genes *STAT3*, *AKT* and *MAPK8* (encoding the JNK protein) were found to be significantly inhibited upon LV application which suggest that the extract interfere with the respective JAK/STAT, MAPK and PI3K/AKT signaling pathways.

Correspondingly, treatment with pure RA in concentrations matching those found in the respective doses of LV extract resulted in similar transcriptional changes. Dose-dependent decrease in the gene expression was registered for *CHUK*, *NFKB1*, *NFKBIA*, *IKBKB*, *RELA* upon RA treatment which was higher than the one achieved by DEXA, but not by LV extract. These results hint that RA is only partly responsible for the LV extract inhibition in NF-κB-related genes. The inflammatory mediators *IL6* and *CCL20* mRNA expression was significantly downregulated by RA in a manner exceeding the effect of LV treatment. Similarly to the LV extract treatment, the gene expression of *STAT3* and *AKT* was influenced by RA, but to a lesser extent. In contrast, RA application did not affected *CCL2* expression. Additionally, the *MAPK8* (encoding JNK) and *MAPK1* (encoding ERK1/2) genes were dose-dependently decreased as a result of RA application. Interestingly, RA treatment in stimulated keratinocytes resulted in significant downregulation of *JAK2* and *STAT1*, which was not observed upon the LV extract application.

Together, these findings indicated that LV extract affects mainly genes from the NF-κB signaling in activated keratinocytes, while RA interfere with JAK/STAT signaling as well.

### *Lavandula angustifolia* Extract and Pure Rosmarinic Acid Disturbs JAK2/STAT1 Signaling in Keratinocytes

**FIGURE 3:**
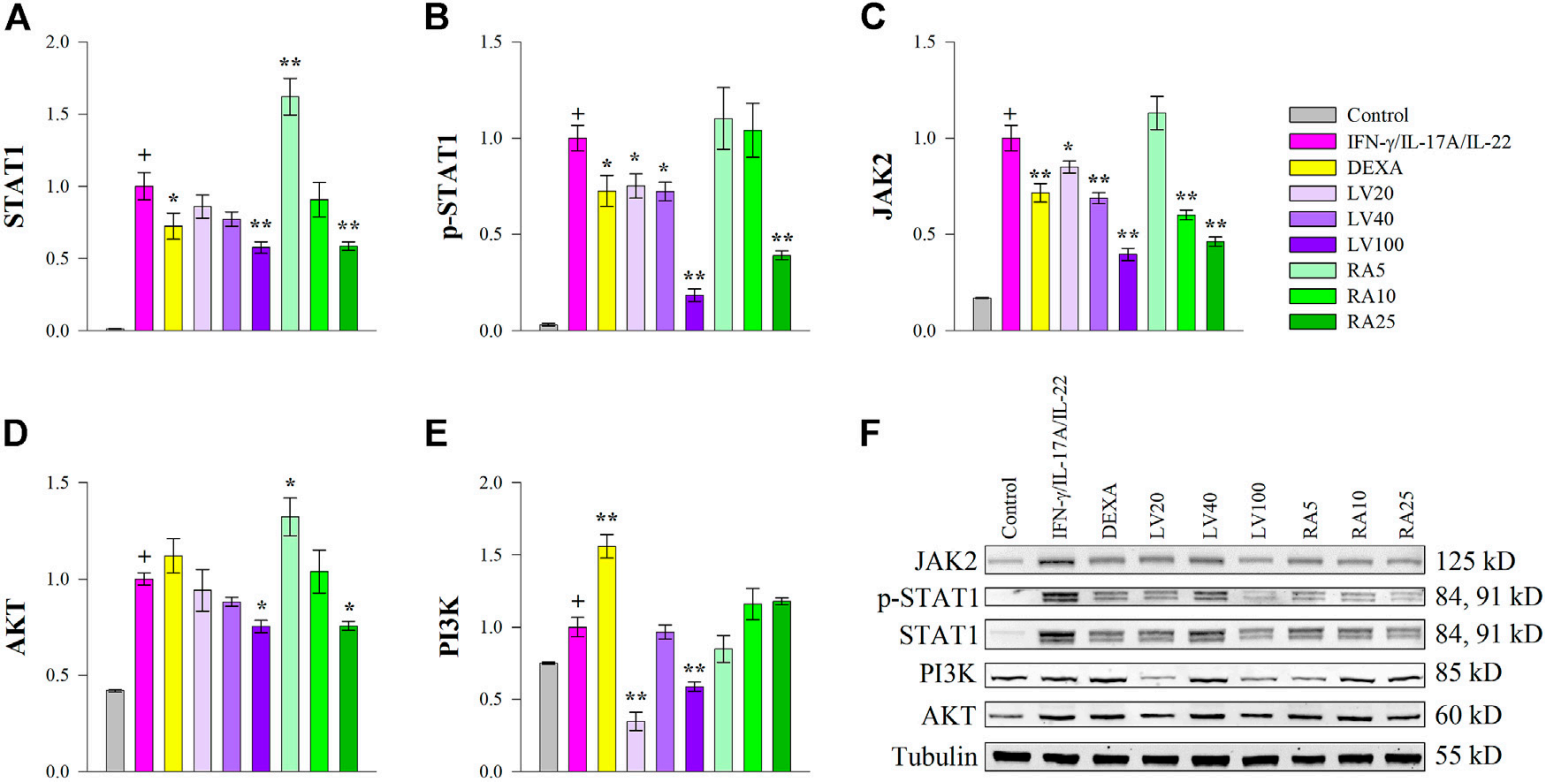
*Lavandula angustifolia* extract (LV) and pure rosmarinic acid (RA) both inhibit JAK2/STAT1 signaling in HaCaT cells. Protein expression of STAT1 **(A)**, phosphorylated STAT1 **(B)**, JAK2 **(C)**, AKT **(D)** and PI3K **(E)** and representative bands from the Western blotting analysis **(F)**. The results are presented as mean ± SEM from three independent experiments. +*p* < 0.05 compared to control cells; *p < 0.05 and **p < 0.01 compared to psoriasis-like model group.

Cytokines such as IFN-γ, IL-17A or IL-22 bind to cell surface receptors and the signal transduction occurs through JAKs, which in turn promotes STAT1 and/or STAT3 phosphorylation and activation in psoriasis (Wolf et al., 2010; Shi et al., 2011; Kim et al., 2014; Zhang et al., 2018; Itoh et al., 2019). As STAT1 expression was activated predominantly over STAT3 by cytokine stimulation in our study, we then evaluated whether RA could antagonize STAT1 activation induced by IFN-γ/IL-17A/IL-22 in keratinocytes at a protein level. The Western blot analysis (Figure 3) showed that the inhibitory effects of both LV extract and RA on the total protein (Figure 3A) and phosphorylation levels of STAT1 (Figure 3B) were in a concentration-dependent manner in HaCaT cells. Further, at the highest concentration used the decrease in STAT1 and p-STAT1 levels were greater than achieved upon DEXA treatment. Moreover, LV extract and RA significantly decreased the total protein levels of JAK2 (Figure 3C) in a concentration dependent manner. The RT-qPCR analysis showed that the JAK2 and STAT1 transcriptional activity was significantly decreased by RA (Figure 2), which corresponded to the changes observed at a protein level. In contrast, *JAK2* and *STAT1* mRNA expression levels (Figure 2) were not influenced upon LV treatment, however, their respective protein levels were significantly diminished. The discrepancy between gene expression and protein abundance could be due to either posttranslational modifications or stimulated protein degradation of JAK2 and/or STAT1 induced by the LV extract.

The PI3K/AKT signaling is involved in cell proliferation and is a hallmark of psoriasis in regard to hyper-keratinization (Engelman et al., 2006; Li et al., 2019). The gene expression analysis revealed that both LV and RA downregulated AKT (Figure 2) to an extent similar to that of the DEXA treatment. In order to acquire further clarification of the mechanism that LV and RA inhibits psoriasis-like keratinocyte activation PI3K/AKT pathway was examined at a protein level. The results indicated that both LV and RA treatments at the highest concentrations used resulted in a moderate decrease in AKT (Figure 3D) while PI3K (Figure 3E) was influenced only by the LV extract.

Together, these results indicated that RA interfere with JAK2/STAT1 signaling in keratinocytes. Further, the biotechnologically produced LV extract rich in RA influence both JAK2/STAT1 and PI3K/AKT signaling pathways.

## Discussion

Chronic inflammation, abnormal proliferation and differentiation of keratinocytes, the most predominant cell type of the *epidermis*, are the key events involved in psoriasis development. Therefore, inhibiting keratinocyte inflammation can be implemented as effective therapeutic approach in the management of psoriasis. In the present study, we demonstrated that IFN-γ/IL-17A/IL-22 stimulation induce psoriasis-like changes in human keratinocytes. Additionally, we provide evidence that psoriasis-related inflammation in keratinocytes could be reversed by LV and RA treatment.

Interplay between activated immune cell and keratinocytes is critical determinant of the pathology of psoriasis, hence, inflammatory cytokines challenge keratinocytes to induce psoriatic phenotype (Federici et al., 2002; Nograles et al., 2008; Nestle et al., 2009; Greb et al., 2016). Stimulation with Th cells-derived cytokines such as IFN-γ activates predominantly STAT1 phosphorylation (Nograles et al., 2008; Antunes et al., 2011; Hald et al., 2013; Albanesi et al., 2018) while IL-17A induce both STAT1 and STAT3 signaling (Nograles et al., 2008; Shi et al., 2011). However, both IFN-γ and IL-17A are known to contribute to psoriasis-related inflammation (Federici et al., 2002; Wolk et al., 2006; Shi et al., 2011). On the other hand, upon stimulation with IL-22 the activated keratinocytes respond through STAT3 up-regulation and changes in their differentiation profile toward hyper-keratinization and hyperproliferation as hallmarks of psoriasis (Wolk et al., 2006; Nograles et al., 2008; Shi et al., 2011). Consequently, exposure of keratinocytes to the IFN-γ/IL-17A/IL-22 cytokine combination represents a reliable psoriasis-like *in vitro* model (Kim et al., 2014; An et al., 2018; Kim et al., 2018; Shi et al., 2018; Zhang et al., 2018; Li et al., 2020).

Until now, very little is known about the roles of RA in keratinocytes. It has been previously reported that RA counteracts UVA-related impairment in murine melanoma cells (Sanchez-Campillo et al., 2009), inhibits IFN-γ-induced inflammation in primary human keratinocytes (Georgiev et al., 2012), protects human fibroblasts from the damage caused by parabens (Matwiejczuk et al., 2020) and stimulates collagen biosynthesis (Chaiyana et al., 2019; Matwiejczuk et al., 2020). In our previous study, RA treatment resulted in downregulated MCP1 and IP-10 levels in IFN-γ-stimulated primary human keratinocytes (Georgiev et al., 2012). Here, we have evaluated the anti-inflammatory potential of LV extract and pure RA in psoriasis-like model in human keratinocytes. The RA content in the LV extract was determined to be 10% in the dry extract. Hence, the biotechnologically produced LV extract could serve as a source of RA production. Moreover, the high RA content in the extract suggest that its bioactivity is predominantly due to the presence of this specific phenolic compound.

The activated keratinocytes respond to immune stimulation in an attempt to resist psoriatic changes with secretion of chemoattractant molecules (such as MCP1 and CCL20), inflammatory cytokines (IL-6, IL-8, IL-4) and antibacterial peptides (S100A; Wolk et al., 2006; Nograles et al., 2008; Andres et al., 2013; Greb et al., 2016; Lee et al., 2019; Liang et al., 2019). However, induced keratinocytes further attract immune cells at the psoriatic lesions and aggravate the inflammatory milieu (Wolk et al., 2006; Nograles et al., 2008; Wolf et al., 2010; Itoh et al., 2019). As expected, IFN-γ/IL-17A/IL-22 stimulation in keratinocytes resulted in high overexpression of *IL6* and *CCL2* that confirmed the induction of psoriatic inflammation. Application of LV extract reverted *CCL2* and *IL6* upregulation. Similarly, RA treatment reduced *IL6* to even greater extent and to levels comparable with DEXA. However, the *CCL2* mRNA expression was not affected upon RA application, which indicated that other compounds are influencing the effect observed by the LV treatment.

The transcriptional activator NF-κB is one of the main involved in the development of the inflammatory response (Wolf et al., 2010; Andres et al., 2013; Schwartz et al., 2017; Bigas et al., 2018). Activation of NF-κB in keratinocytes is found in psoriatic lesions and is known to contribute to amplified inflammatory response reflected by elevated production of immune-related proteins such as CCL20 and S100A7 (Kim et al., 2018; Zhang et al., 2018). In the present study, key genes responsible for the NF-κB activation were investigated, namely, *CHUK*, *IKBKB*, *NFKBIA*, *NFKB1* and *RELA*. Stimulation with the cytokine combination in HaCaT cells led to elevation in all NF-κB-related genes, consistent with a role in inducing psoriasis-like inflammation. Glucocorticoid therapy acts predominantly through NF-κB inhibition to produce its potent anti-inflammatory effect (Bigas et al., 2018). Therefore, downregulation of the expression of NF-κB-related genes, as well as that of its downstream targets such as *IL6*, *CCL2* and *CCL20* upon DEXA treatment in keratinocytes was expected. Interestingly, both LV extract and pure RA downregulated all studied genes of the NF-κB pathway. Suppression of NF-κB signaling upon RA treatment was reported previously in human leukemic monocytes U937 (Chaiyana et al., 2019) and in a human monocytic cell line THP-1 (Leu et al., 2019), and predicted to be responsible for the anti-inflammatory effect of RA. Correspondingly, our results suggest that RA is the most probable compound responsible for the LV extract activity toward NF-κB.

The PI3K/AKT signaling is activated in psoriatic keratinocytes and reflect cell growth promotion, induction of hyperproliferation and hyper-keratinization (Engelman et al., 2006; Li et al., 2019; Thatikonda et al., 2020). Data from the gene expression analysis indicated that both LV extract and pure RA downregulated *AKT*. However, pure RA treatment reduced AKT protein level only at the highest concentration used. The LV extract influenced both PI3K and AKT significantly at a protein level. We speculate that the interaction of LV with PI3K/AKT signaling could be due to the phytochemical complexity of the extract, and thus the impact of other bioactive compounds besides RA. The modulation of PI3K/AKT observed upon LV treatment is worth further in-depth investigation as it could lead to reduced proliferation in psoriatic keratinocytes and, consequently, reduce lesion size.

The IFN-γ, IL-17A and IL-22 are key pathogenic cytokines in psoriasis and one of the main mediators of cytokine signaling is STAT1 (Wolk et al., 2006; Nograles et al., 2008; Antunes et al., 2011; Shi et al., 2011; Hald et al., 2013). Additionally, elevated levels of phosphorylated STAT1 are found in psoriatic lesions (Federici et al., 2002; Antunes et al., 2011; Hald et al., 2012). The JAKs belong to the group of cytoplasmic tyrosine kinases that are essential upstream STAT activators (Andres et al., 2013; Kim et al., 2014; Schwartz et al., 2017). Therefore, inhibition of JAKs could abolish STAT activation induced by cytokines. Recently JAK inhibitors have been proposed as attractive therapeutics for psoriasis (Schwartz et al., 2017). Our results demonstrated that STAT1 emerges as a key target of both LV extract and its main bioactive component RA, through which they exert the anti-inflammatory effect in psoriasis-like model in keratinocytes. Here, we provide evidence that both LV extract and pure RA effectively reverted STAT1 phosphorylation to levels exceeding that of DEXA, suggesting that RA could block the STAT1 signaling in keratinocytes. Additionally, to further clarify the underlying mechanisms, we have evaluated JAK2 protein abundance. We identified that LV and RA reduced JAK2 protein levels in keratinocytes, suggesting that the inhibition of STAT1 activation is achieved through inhibition of JAK2. It is worth mentioning that previous studies showed that RA inhibited the IL-4 and IFN-γ expression activated CD4^+^ T cells and de-creased immune cell infiltration in atopic dermatitis skin lesions in mice (Jang et al., 2011). Therefore, RA could inhibit inflammation in both immune cells and keratinocytes that are implicated in the development of chronic inflammatory skin conditions such as psoriasis.

As RA has been shown to be relatively safe to normal cells in the majority of laboratory settings (Domitrovic et al., 2014; Liang et al., 2016; Shang et al., 2016; Nunes et al., 2017; Chen et al., 2018; Joardar et al., 2019; Leu et al., 2019; Luo et al., 2020; Vasileva et al., 2021), our results here suggest that RA is a potential agent for combating psoriasis. The moderate bioavailability of RA upon oral administration is usually a drawback for its utilization in drug therapy (Nunes et al., 2017; Shen et al., 2017; Luo et al., 2020). On the other hand, RA topical application is found to be effective in skin disorders models such as atopic dermatitis model in mice (Jang et al., 2011). Anti-psoriatic preparations are most preferably used topically, such that RA and RA-rich extracts such as LV could be successfully integrated in products for psoriasis management.

**FIGURE 4:**
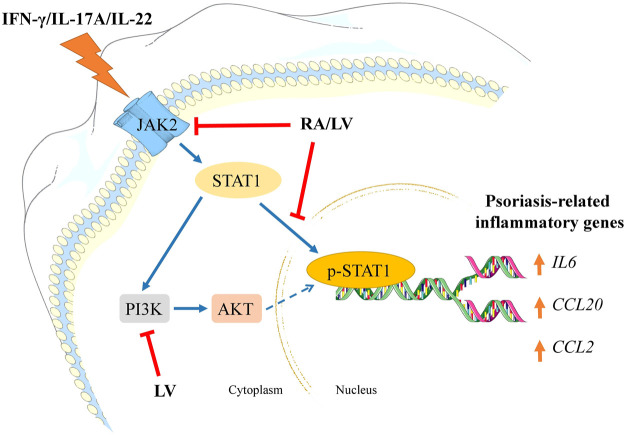
Schematic representation of the proposed mechanisms of action of *Lavandula angustifolia* extract (LV) and rosmarinic acid (RA) in psoriasis-like inflammation model in human keratinocytes. HaCaT cells exposed to IFN-γ/IL-17A/IL-22 respond with activation of JAK2/STAT1 and PI3K/AKT signaling pathways. Activation of JAK2 upon cytokine stimulation leads to STAT1 activation and its subsequent phosphorylation. Following nuclear translocation the phosphorylated STAT1 induces transcriptional activation of psoriasis-related inflammatory genes (*e.g., IL6*, *CCL20*, *CCL2*). Activated PI3K/AKT axis in psoriatic keratinocytes correlates with induction of hyperproliferation and aggravation of the inflammatory milieu. In the present investigation, both RA and LV inhibited JAK2 and diminished STAT1 phosphorylation, hence, preventing inflammatory activation. Additionally, the LV extract disrupted PI3K/AKT signaling which could contribute to decrease in proliferation rate in activated keratinocytes. Taken together, the obtained data suggest that LV and RA possess inhibitory effect on psoriasis-related inflammation in human keratinocytes.

In summary, our findings demonstrate that the biotechnologically produced LV extract diminished psoriasis-like inflammation in human keratinocytes by interfering the JAK2/STAT1 signaling and its effectiveness is due to the high content of RA. Further, both LV extract and pure RA downregulated NF-κB-related genes that are overexpressed in psoriatic condition. We also revealed that LV could influence PI3K/AKT signaling suggesting potential modulation of keratinocyte proliferation in psoriasis (Figure 4). Collectively, these results suggest that the biotechnologically produced LV extract or pure RA may serve as promising therapeutic alternatives in psoriasis management. Further *in vivo* validation is needed to confirm their effectiveness in the therapy of psoriasis.

## Data Availability

The original contributions presented in the study are included in the article/Supplementary Material, further inquiries can be directed to the corresponding author.
