# Supplementary material for: Biotechnologically Produced Lavandula angustifolia Mill. Extract Rich in Rosmarinic Acid Resolves Psoriasis-Related Inflammation Through Janus Kinase/Signal Transducer and Activator of Transcription Signaling
Source: Front Pharmacol. 2021 Apr 27;12:680168. doi: 10.3389/fphar.2021.680168 (PMC8111009; doi:10.3389/fphar.2021.680168)
Supplement: Supplementary file 1 [file DataSheet1.docx]

Supplementary Material

**Biotechnologically-produced *Lavandula angustifolia* Mill. Extract Rich in Rosmarinic Acid Resolves Psoriasis-related Inflammation Through JAK/STAT Signaling**

**Ivanka K. Koycheva^1,2^, Liliya V. Vasileva^2^, Kristiana M. Amirova^1,2^, Andrey S. Marchev^1,2^, Zhivka P. Balcheva-Sivenova^1,2^, Milen I. Georgiev^1,2*^**

^1^ Laboratory of Metabolomics, Department of Biotechnology, The Stephan Angeloff Institute of Microbiology, Bulgarian Academy of Sciences, Plovdiv, Bulgaria

^2^ Department Plant Cell Biotechnology, Center of Plant Systems Biology and Biotechnology, Plovdiv, Bulgaria

*** Correspondence:**Milen I. Georgiev
milengeorgiev@gbg.bg

**
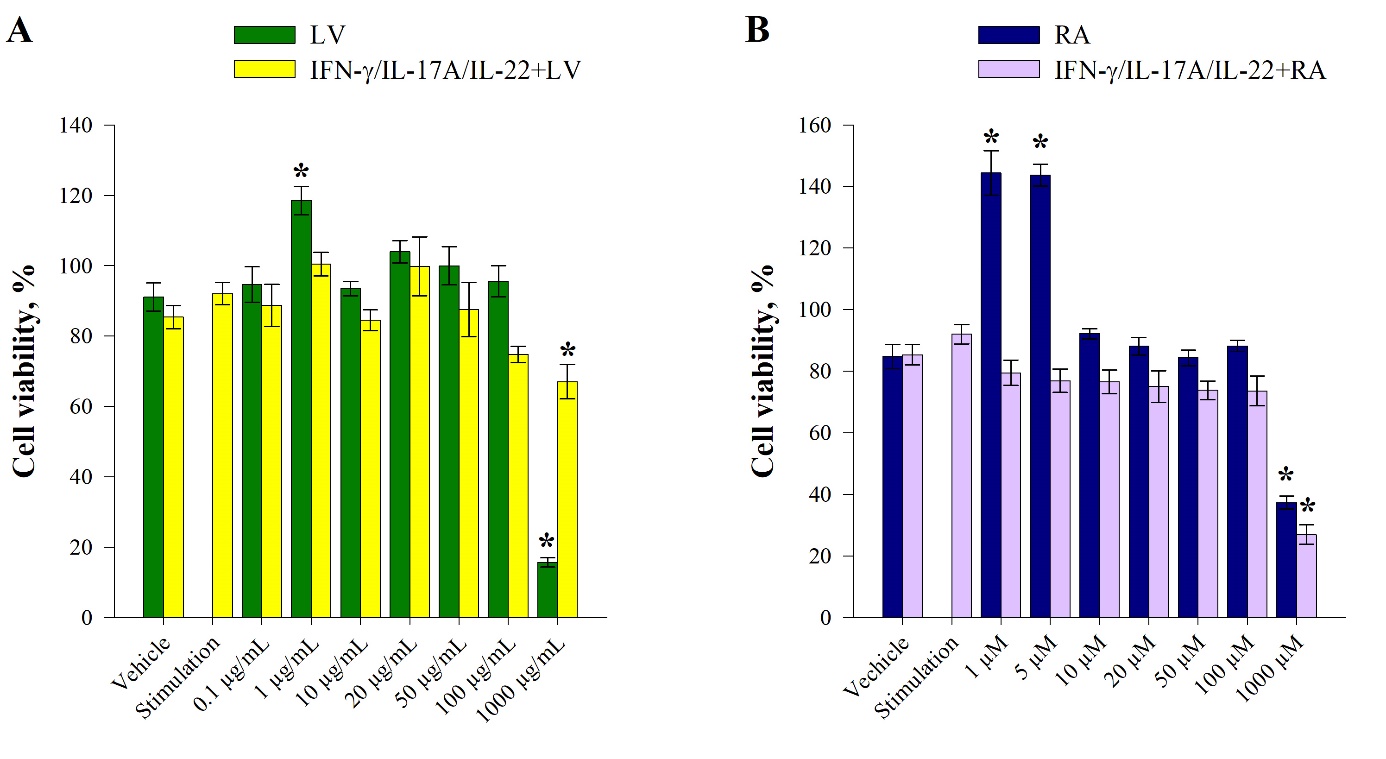
**

**Supplementary Figure S1.**  *Lavandula angustifolia* extract (LV; **A**) and pure rosmarinic acid (RA, **B**) effect on cell viability in human keratinocytes. Both LV and RA at the 24th of treatment did not affect cell viability in HaCaT cells up to 100 μg/mL and 100 μM respectively.

Cell viability was evaluated through MTT assay. Shortly, HaCaT cells (1 x 10^4^ cells/well) were seeded in 96-well plates and were cultured for 24 h to reach confluence. Then cells were treated with LV (0.1, 1, 10, 20, 50, 100, 500, 1000 µg/mL) or RA (0.1, 1, 5, 10, 20, 50, 100, 1000 µM) with or without IFN-γ/IL-17A/IL-22 stimulation (1/1/1 ng/mL). On the 24th hour of treatment 10 μL MTT reagent (5 µg/mL) per well was added and left for 3 h of incubation at 37 0C. Finally, 200 μL of 5% formic acid in isopropanol was used to dissolve the purple formazan crystals formed within the viable cells. Absorbtion was measured on a microplate reader Antos Zenyth 340 (Biochrom Ltd, Cambridge, United Kingdom) at 570 nm with reference filter at 620 nm. Cell viability was expressed as percentage from the non-treated controls, mean±SEM and *p<0.05 compared to non-treated controls.

**Supplementary Table S1:** Primer sequences for the RT-qPCR analysis.

| **Target gene (human)** | **Sequence forward primer (5’ - 3’)** |  | **Sequence reverse primer (5’ - 3’)** |
| --- | --- | --- | --- |
| *AKT1* | CGAGCTGTTCTTCCACCTGT |  | TAATGTGCCCGTCCTTGTCC |
| *CCL2* | GATCTCAGTGCAGAGGCTCG |  | TTTGCTTGTCCAGGTGGTCC |
| *CCL20* | AGTTGTCTGTGTGCGCAAATCC |  | TCCAACCCCAGCAAGGTTCT |
| *CHUK* | TTCTGTTACCACCTGATGAAAGTCT |  | ATTGAGAGGCTGGTTTCCGAG |
| *GAPDH* | CCCACTCCTCCACCTTTGAC |  | TCCTCTTGTGCTCTTGCTGG |
| *JAK2* | CAAAGCAACTGTCATGGCCC |  | TCTCGCTCGACAGCAAAAGT |
| *IL6* | TGCAATAACCACCCCTGACC |  | GTGCCCATGCTACATTTGCC |
| *IKBKB* | TGAGAAGACTGTTGTCCGGC |  | CACTCTTCTTGGCTGGCTCA |
| *MAPK8* | CTGAAGCAGAAGCTCCACCA |  | CCTGTGCTAAAGGAGAGGGC |
| *MAPK1* | CGTGTTGCAGATCCAGACCA |  | CCTGGAAAGATGGGCCTGTT |
| *MAPK14* | GGGTTACGTGTGGCAGTGAA |  | CCCATGAGATGGGTCACCAG |
| *NFKB1* | GGCTACACCGAAGCAATTGAA |  | CAGCGAGTGGGCCTGAGA |
| *NFKBIA* | GAAGTGATCCGCCAGGTGAA |  | CTCACAGGCAAGGTGTAGGG |
| *RELA* | TTCCAACTGCCCCCAACTTT |  | TTTGAGTTTCCCCAGCTCCC |
| *S100A7* | ACACTCAAGCTGAGAGGTCCA |  | AAGACATCGGCGAGGTAATTTGT |
| *STAT1* | GGATCAGCTGCAGAACTGGT |  | GAAGGTGCGGTCCCATAACA |
| *STAT3* | ACCAACGACCTGCAGCAATA |  | TCTGCAGCTTCCGTTCTCAG |
| *TUBB* | AGCCGTCTTACTCAACTGCC |  | GTCACCCAGAATGGCAGAA |
